# A Comprehensive Analysis of COVID-19 Misinformation, Public Health Impacts, and Communication Strategies: Scoping Review

**DOI:** 10.2196/56931

**Published:** 2024-08-21

**Authors:** Sezer Kisa, Adnan Kisa

**Affiliations:** 1 Department of Nursing and Health Promotion Faculty of Health Sciences Oslo Metropolitan University Oslo Norway; 2 Department of Health and Exercise School of Health Sciences Kristiania University College Oslo Norway; 3 Department of International Health and Sustainable Development School of Public Health and Tropical Medicine Tulane University New Orleans, LA United States

**Keywords:** communication strategies, COVID-19, infodemic, misinformation, public health

## Abstract

**Background:**

The COVID-19 pandemic was marked by an *infodemic*, characterized by the rapid spread of both accurate and false information, which significantly affected public health. This infodemic led to confusion, mistrust in health authorities, noncompliance with health guidelines, and engagement in risky health behaviors. Understanding the dynamics of misinformation during the pandemic is crucial for developing effective public health communication strategies.

**Objective:**

This comprehensive analysis aimed to examine the complexities of COVID-19 misinformation. Specifically, it sought to identify the sources and themes of misinformation, the target audiences most affected, and the effectiveness of various public health communication strategies in mitigating misinformation.

**Methods:**

This scoping review used the MEDLINE (PubMed), Embase, and Scopus databases to identify relevant studies. An established, methodical framework for scoping reviews was used to review literature published between December 2019 and September 2023. The inclusion criteria focused on peer-reviewed studies published in English that address COVID-19 misinformation and its sources, themes, and target audiences, as well as the effectiveness of public health communication strategies.

**Results:**

The scoping review identified that misinformation significantly impacted mental health, vaccine hesitancy, and health care decision-making. Social media and traditional media were major conduits for spreading misinformation. Key misinformation themes included the origins of the virus, ineffective treatments, and misunderstandings about public health measures. Misinformation sources ranged from social media platforms to traditional media outlets and informal networks. The impact of misinformation was found to vary across different regions and demographic groups, with vulnerable populations being disproportionately affected. Effective strategies to counter misinformation included enhancing health literacy; using digital technology; promoting clear, authoritative communication; and implementing fact-checking mechanisms. In addition, community engagement and targeted health campaigns played a crucial role in addressing misinformation.

**Conclusions:**

The review emphasizes the critical need for accurate and consistent messaging to combat misinformation. Cooperative efforts among policy makers, health professionals, and communication experts are essential for developing effective interventions. Addressing the infodemic is vital for building a well-informed, health-literate society capable of handling misinformation in future global health crises. The study provides valuable insights into the dynamics of misinformation and highlights the importance of robust public health communication strategies. These findings can guide future efforts to mitigate the impact of misinformation during health emergencies.

## Introduction

### Background

The COVID-19 pandemic, a health crisis of unprecedented scale in the 21st century, was accompanied by an equally significant and dangerous phenomenon—an *infodemic*. The World Health Organization defines an infodemic as the rapid spread and overabundance of information—both accurate and false—that occurs during an epidemic [1]. A tidal wave of misinformation, disinformation, and rumors characterized the infodemic during the COVID-19 pandemic. This led to widespread confusion, mistrust in health authorities, noncompliance with health guidelines, and even risky health behaviors [2-4].

Moreover, the role of political leaders in shaping the narrative around COVID-19 policies significantly influenced these dynamics. In countries such as the United States, Brazil, and Turkey, the intersection of political ideology and crisis management led to increased societal polarization. Leaders in these nations used communication strategies ranging from denying the severity of the pandemic to promoting unproven treatments [5,6]. This complex interplay between leadership communication and public response underscores the critical need for science-based policy communication and the responsible use of social media platforms to combat misinformation and foster societal unity in the face of a global health crisis.

Furthermore, the emergence of the COVID-19 infodemic highlighted the crucial role of social media literacy in combating misinformation. Educating the public on discerning credible information on the web has emerged as a pivotal strategy for mitigating the spread of misinformation and its consequences [7].

Misinformation during public health crises has been a recurring problem. Historical examples from the Ebola outbreak, such as rumors that the virus was a government creation or that certain local practices could cure the disease, highlight how misinformation can hinder public health responses [8]. False beliefs, such as that drinking salt water would cure Ebola or that the disease was spread through the air, led to a mistrust of health workers and avoidance of treatment centers, exacerbating the crisis [9]. In the context of COVID-19, misinformation was particularly pervasive, with false claims about the effectiveness of various nostrums, leading to panic buying and shortages [3,10]. The impact of such misinformation varied across regions [4,11]. These dynamics were often fueled by psychological and social factors, including fear, uncertainty, and the reinforcing nature of social media algorithms, which created echo chambers of false information [12,13]. The wide-ranging consequences affected not only immediate health behaviors but also the trust in, and response to, public health authorities [2,14].

Misinformation during a public health crisis is nothing new. However, the scale and speed at which misinformation spread during the COVID-19 pandemic are unparalleled. This situation was exacerbated by the widespread use of social media and the internet, where rumors can rapidly reach large audiences [9,15]. This spread of misinformation had far-reaching consequences: it undermined public health efforts, promoted harmful practices, contributed to vaccine hesitancy, and possibly prolonged the pandemic [8,10,12,16]. These effects went beyond individual health behaviors; they influenced public health policies and diminished trust in health authorities and the scientific community [12,17,18].

In light of these challenges, the machine learning–enhanced graph analytics (MEGA) framework has emerged as a novel approach to managing infodemics by leveraging the power of machine learning and graph analytics. This framework offers a robust method for detecting spambots and influential spreaders in social media networks, which is crucial for assessing and mitigating the risks associated with infodemics. Such advanced tools are essential for public health officials and policy makers to navigate the complex landscape of misinformation and to develop more effective communication strategies [19]. Furthermore, combating this infodemic necessitates a strategic approach encapsulating the “Four Pillars of Infodemic Management”: (1) monitoring information (infoveillance) to track the spread and impact of misinformation; (2) enhancing eHealth literacy and science literacy, empowering individuals to evaluate information critically; (3) refining knowledge quality through processes such as fact checking and peer review, ensuring the reliability of information; and (4) ensuring timely and accurate knowledge translation, minimizing the distortion by political or commercial interests [20]. These measures are essential for mitigating the impact of misinformation and guiding the public and professionals toward quality health information during the pandemic and beyond. The COVID-19 pandemic has highlighted the need for improved public health communication and preparedness strategies, particularly in countering misinformation to prevent similar challenges in future health crises [15,21].

### Pertinent Questions

Recognizing the unique challenges posed by the COVID-19 infodemic, this comprehensive scoping review seeks to systematically explore various dimensions of misinformation related to the pandemic. Our investigation is informed by a critical analysis of existing literature, noting a gap in studies that collectively examine the themes, sources, target audiences, impacts, interventions, and effectiveness of public health communication strategies against COVID-19 misinformation. To the best of our knowledge, this is the first review that attempts to bridge this gap by providing a comprehensive and integrated analysis of these key dimensions. While individual aspects of misinformation have been addressed in prior research, there lacks a comprehensive review that integrates these components to offer a holistic understanding necessary for effective countermeasures. Therefore, our review is structured around four pertinent questions, each carefully selected for their significance in advancing our understanding of the COVID-19 infodemic and its counteraction:

1. What is the extent of COVID-19 misinformation? How can it be addressed?
2. What are the primary sources of COVID-19 misinformation?
3. Which target audiences are most affected by COVID-19 misinformation?
4. What public health communication strategies are being used to combat COVID-19 misinformation?

These questions were selected to emphasize critical areas of COVID-19 misinformation that, when addressed, can significantly contribute to bridging technical and knowledge gaps in our response to current and future public health emergencies. By detailing our study’s contributions to existing literature, we aim to present distinctive understandings crucial for policy makers, health professionals, and the public in effectively addressing misinformation challenges.

## Methods

This scoping review was conducted following the methodology framework defined by Arksey and O’Malley [16] and elaborated upon by Levac et al [17]. This framework, recognized for its systematic approach, involves five stages: (1) defining the research question; (2) identifying relevant studies; (3) selecting appropriate literature; (4) charting the data; and (5) collating, summarizing, and reporting the results.

### Databases and Search Strategies

The literature search targeted 3 major databases: MEDLINE (PubMed), Embase, and Scopus. These databases were selected for their comprehensive coverage of medical, health, and social science literature. The search strategy was crafted using a combination of keywords and subject headings related to COVID-19, misinformation, and public health communication. We used (“COVID-19” OR “SARS-CoV-2” OR “Coronavirus”) AND (“Misinformation” OR “Disinformation” OR “Fake news” OR “Infodemic”) AND (“Public health outcomes” OR “Health impacts”) AND (“Communication strategies” OR “Public health communication”).

### Eligibility Criteria

The inclusion and exclusion criteria are presented in Textbox 1.

**Inclusion and exclusion criteria.**

**Inclusion criteria**

- Article type: peer-reviewed studies
- Language: published in English
- Publication date: published between December 1, 2019, and September 30, 2023
- Focus: addresses COVID-19 misinformation and its sources, themes, and target audiences, as well as the effectiveness of public health communication strategies
- Study design: empirical studies (eg, cross-sectional, observational, randomized controlled trials, qualitative, and mixed methods)

**Exclusion criteria**

- Article type: non–peer-reviewed articles, opinion pieces, and editorials
- Language: published in languages other than English
- Publication date: published before December 1, 2019, or after September 30, 2023
- Focus: does not address COVID-19 misinformation or its related aspects
- Study design: case studies and anecdotal reports

### Study Selection Process

The study selection process involved an initial screening of titles and abstracts to eliminate irrelevant studies, followed by a thorough full-text review of the remaining articles. This critical stage was conducted by the authors, each with expertise in public health communication and health services research, thereby enhancing the thoroughness and reliability of the selection process. In cases of disagreement, the reviewers engaged in discussions until a consensus was reached on the inclusion of each article. In addition, we adhered to the PRISMA-ScR (Preferred Reporting Items for Systematic Reviews and Meta-Analyses extension for Scoping Reviews) guidelines [18] to enhance the thoroughness and transparency of our review (see Multimedia Appendix 1 for the PRISMA-ScR checklist).

## Results

### Overview

**Figure 1:**
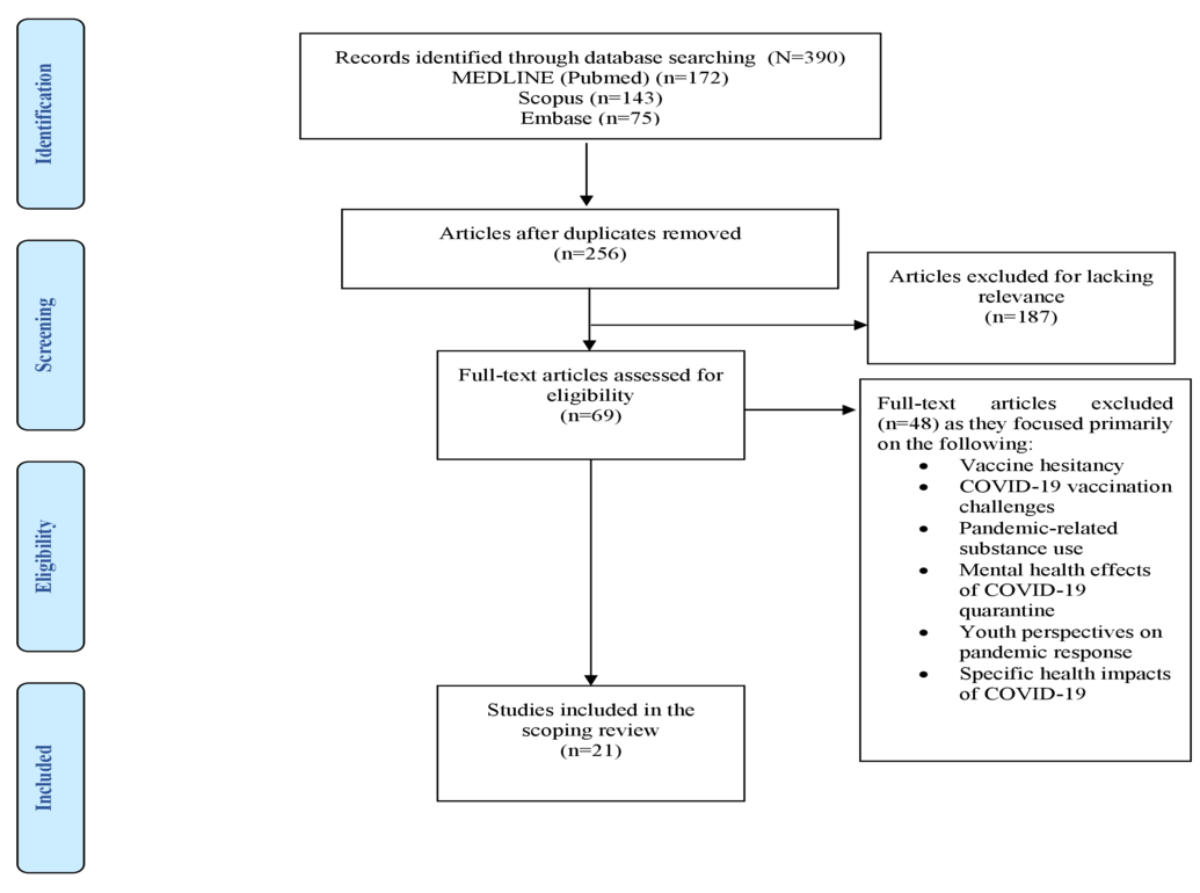
PRISMA (Preferred Reporting Items for Systematic Reviews and Meta-Analyses) flow diagram of the search and screening results.

A total of 390 articles were identified from the 3 databases, of which, after removing 134 (34.4%) duplicates, 256 (65.6%) articles remained. Of these 256 articles, 69 (27%) were selected based on abstract searches. Of the 69 full-text articles, 27 (39%) were assessed for eligibility. Of these 27 studies, 21 (78%) were included in the scoping review (Figure 1). This analysis of the 21 studies provides a comprehensive overview of the many impacts of misinformation during the COVID-19 pandemic, including its characteristics, themes, sources, effects, and public health communication strategies.

### Study Characteristics

The included studies exhibited considerable diversity in terms of their methodologies, geographic focus, and objectives (Table 1). Verma et al [15] conducted a large-scale observational study in the United States, analyzing social media data from >76,000 users of Twitter (subsequently rebranded X) to establish a causal link between misinformation sharing and increased anxiety. By contrast, Loomba et al [11] carried out a randomized controlled trial in both the United Kingdom and the United States to examine the impact of misinformation on COVID-19 vaccination intent across different sociodemographic groups. In the United States, Bokemper et al [22] used randomized trials to assess the efficacy of various public health messages in promoting social distancing. Xue et al [23] used observational methods to explore public attitudes toward COVID-19 vaccines and the role of fact-checking information on social media. These studies collectively used quantitative analysis, web-based surveys, cross-sectional studies, and social network analysis, reflecting the diversity of research approaches. Sample sizes ranged from hundreds to tens of thousands of participants, providing a broad view of the infodemic’s impact. Notably, most of the studies (17/21, 81%) were conducted on the web, underlining the infodemic’s digital nature. The outcomes assessed various public health aspects, including mental health, communication effectiveness, and behavior change. Kumar et al [13] used social network and topic modeling analyses to gain insights into public perceptions on Reddit, contributing to the methodological diversity within the reviewed literature.

**Table 1:** General characteristics of the included studies, misinformation themes, sources of misinformation, and target audience.

Study, year; country; method	Aim	Sample, n	Study outcomes	Misinformation themes	Sources of misinformation	Target audience
Datta et al [24], 2020; India; cross-sectional study	Explore COVID-19 information sources among health care professionals	778 adults	High misinformation prevalence, mainly from social media	Incorrect COVID-19 treatments, false diagnoses, virus transmission misconceptions, public health inaccuracies	Social media, family, WhatsApp, television, friends	Health care professionals in India, implications for the general public
Moscadelli et al [3], 2020; Italy; observational study	Measure circulation of fake and verified COVID-19 news	2102 articles shared on social media platforms	Fake news shared 2.35 million times (23.1% of total shares)	Accidental laboratory release, SARS-CoV-2 origin, bioweapon claims, HIV vaccine, vitamin C and D, 5G technology, consuming garlic	Internet articles, social media platforms	General public, especially susceptible individuals
Hou et al [10], 2020; 12 countries; cross-sectional study	Assess public awareness and behavioral responses to COVID-19	Global public responses to COVID-19 from platforms such as Google Trends and Baidu Index	Public response trends, impact of rumors, communication effectiveness	Traditional Chinese medicines, garlic for prevention, antimalarial treatments	Internet rumors, newspapers, political leaders	Global general public; focus on China, the United States, and traditional medicine regions
Agley et al [14], 2021; United States; randomized controlled trial	Examine effects of exposure to infographic on trust in science and COVID-19 misinformation beliefs	1017 adults	Small trust increase, indirect effect on misinformation mediated by trust	5G transmission, laboratory origin, exaggerated deaths, face mask misinformation	Social media, news outlets, other media	American general public
Teovanović et al [25], 2020; Serbia; cross-sectional survey	Explore irrational beliefs and health behaviors during the COVID-19 pandemic	407 participants	Irrational beliefs predict guideline adherence, pseudoscientific practices, and vaccination intentions	Conspiracy theories, pseudoscientific practices, COVID-19 myths, vaccine-related myths	Digital media outlets	Serbian adults, broader audience susceptible to misinformation
Nowak et al [4], 2021; Poland; cross-sectional survey	Assess COVID-19 preventive behaviors, fears, and conspiracy beliefs	1380 adults	Challenges in adherence, influence of misinformation, fear impact	5G technology as pandemic cause, Chinese government conspiracy, pharmaceutical companies’ profit motives	Web-based social media, message boards	General public, conspiracy theory believers
Loomba et al [11], 2021; United Kingdom and United States; randomized controlled trial	Measure COVID-19 vaccine misinformation impact on intent	8001 adults	Change in intent due to misinformation	Vaccine importance and safety, 5G links, vaccine trial deaths, pandemic conspiracy theories	Social media, various web-based sources	British and American general public
Scholz et al [21], 2021; Germany; cross-sectional survey	Investigate implementation of quarantine measures	562 adults	Improved understanding and community acceptance of quarantine measures	Quarantine measures, health information	Informal channels, possibly social media	General public, adults not frequently engaging with official channels
Ghaddar et al [2], 2022; Lebanon; cross-sectional study	Explore trust in social media, misinformation, and vaccination intent	2653 adults	Vaccination intent, fake news exposure, trust, and conspiracy beliefs	COVID-19 transmission modes, conspiracy theories, medication effectiveness	WhatsApp, Facebook, television and radio, social media platforms	General population of Lebanon
Kim et al [26], 2022; United States; cross-sectional and observational study	Analyze predictors of belief in COVID-19 misinformation on Facebook	6518 adults	Predictors of belief in misinformation, effects on behaviors, correction strategies	Transmission modes, miracle cures, antivaccine beliefs, political conspiracies	User-generated content on Facebook	General public, especially those exposed to misinformation
Huang et al [27], 2022; China; cross-sectional study	Investigate COVID-19 vaccine hesitancy predictors	4289 adults	Sociodemographic predictors, hesitancy reasons, information sources	Vaccine hesitancy due to negative information	Social media, websites, media outlets	Students, health professionals, workers, general population
Bokemper et al [22], 2022; United States; randomized controlled trial	Test public health message effectiveness on social distancing	3184 adults	Impact on distancing beliefs and intentions	COVID-19 conspiracy theories, severity skepticism, downplaying public health measures	Social media, informal networks, public figures	Individuals valuing personal liberty, government mandate opponents
Kumar et al [13], 2022; United States; quantitative observational study	Analyze perceptions of COVID-19 vaccines on Reddit	266,840 Reddit posts	Vaccine-related events and public attitudes	Vaccine efficacy doubts, conspiracy theories, skepticism regarding science and media	Reddit user posts in subreddits	Reddit users, antivaccine subreddit frequent users
Xue et al [23], 2022; United States; observational study	Investigate attitudes toward COVID-19 vaccines on Facebook	12,553 Facebook posts	Public attitude shifts, fact-checking effectiveness	Vaccine efficacy questions, safety views, effectiveness challenges, misinterpretation, emotional manipulation	Politicians, social media, health institutions	General public, vaccine-hesitant people, pro- and antivaccine groups
Mourali and Drake [28], 2022; United States; randomized web-based experiment	Assess social media debates on masking and COVID-19 misinformation	500 adults	Attitude, belief, behavior changes from debates	Masking efficacy, truth objectivity, antimask arguments	Reddit thread: user *citizen-health* versus user *Health_Scientist*	General public, online forum users, conspiracy-prone individuals
Verma et al [15], 2022; United States; observational study	Study Twitter (subsequently rebranded X) misinformation impact on anxiety	76,985 Twitter users	Causal link between misinformation sharing and anxiety	Vitamins, gargling, 5G technology, involvement of Bill Gates	Twitter	General Twitter users, vulnerable US women, racial minority individuals
AL-Jalabneh [29], 2023; Jordan; qualitative study	Explore vaccine hesitancy due to misinformation	30 vaccine-hesitant adults	Role of misinformation in increased vaccine hesitancy, safety, and effectiveness concerns	Social media misinformation, conspiracy theories, safety doubts, vaccine distrust	Social media (Facebook and WhatsApp), influencers, foreign health experts	Jordanian citizens, active social media users
Gruzd et al [30], 2023; Canada; observational study	Examine Facebook and YouTube for vaccine misinformation	539 YouTube videos shared on Facebook	Prevalence and nature of vaccine misinformation	Vaccine safety, efficacy, ingredients, conspiracy theories	Facebook groups and pages, YouTube videos	Facebook and YouTube users
Kim et al [12], 2023; United States; cross-sectional survey	Investigate impact of misinformation on trust and compliance	1400 adults	Misinformation linked to lower trust in health experts, guideline compliance	False claims about prevention, treatment, severity	Politicians, media, social networks	American adult population
Kosiyaporn et al [31], 2023; Thailand; mixed methods study	Assess vaccine acceptance factors	193,744 adults	Factors influencing vaccine acceptance: public perceptions, attitudes	Vaccine efficacy, side effects, immunity misconceptions	Lack of trust in media, government, celebrities, social media; trustworthy: health professionals, academics	General population, those with limited access to reliable information
Ugarte and Young [32], 2023; United States; randomized controlled trial	Address misinformation, vaccine hesitancy among essential workers	120 adults	Reduced misinformation beliefs, increased vaccine information requests	COVID-19 vaccine misinformation, natural immunity	Social media (Facebook groups), non–peer-reviewed studies	American essential workers, vaccine-hesitant people

### Misinformation Themes and Sources

#### Misinformation Themes

The results of the studies reported many themes that presented a diverse and interconnected landscape of COVID-19 misinformation. A significant amount of this misinformation related to the virus’s origins and transmission, with theories varying from accidental laboratory releases to purported links with 5G technology. These theories often reflected a tendency to misinterpret scientific data or attribute the pandemic to external and frequently sensational causes (Table 1) [3,4,14].

A significant proportion of misinformation concerned treatments and preventives for COVID-19, where unscientific remedies (accidental or deliberate) and vitamin supplements were touted as effective [10,15,24]. This was coupled with widespread misconceptions and conspiracy theories about COVID-19 vaccines [11,13,27,29,30].

Public health measures such as the effectiveness of masks and social distancing were often mischaracterized or misrepresented, sometimes due to political and economic theories [22,25,28]. Social media played a significant role in amplifying dangerous beliefs and practices [12,29]. The studies demonstrate that misinformation during the pandemic ranged from basic misunderstandings to elaborate conspiracy theories [2,21,23,27,31,32].

#### Sources of Misinformation

The studies provide a comprehensive analysis of the various sources of COVID-19 misinformation, with a particular focus on social media platforms such as Facebook, WhatsApp, Twitter, Reddit, and YouTube, which were repeatedly identified as primary channels for spreading false information (Table 1) [2-4,11-14,22,24,26,27,29,30,32]. These platforms not only facilitated the spread of misinformation through user-generated content but also through public figures and political leaders, whose remarks often fueled rumors and unsubstantiated claims [10,23,31]. Traditional media sources, including television, newspapers, and radio, also added to the misinformation landscape, either by directly spreading false information or by passing on misleading statements and rumors [2,15]. The influence of informal networks, such as family, friends, and community gatherings, was highlighted, pointing to the significance of word-of-mouth communication in the dissemination of misinformation [21,22,24]. Furthermore, the studies identified specific web-based communities and forums, such as Facebook groups and subreddits, where misinformation was not only shared but also reinforced within echo chambers [13,28,32].

#### Target Audience of Misinformation

The selected studies revealed a complex landscape of COVID-19 misinformation targeting diverse audiences, with a significant focus on the general public across countries; for instance, Datta et al [24] and Hou et al [10] identified both health care professionals and the broader global population, including those in China, the United States, and countries with traditional medicine practices, as key recipients of misinformation (Table 1). Susceptibility to misinformation was also observed in individuals with low health literacy, depression, or susceptibility to conspiracy theories [3,4,13,25] or vaccine-hesitant individuals and those with a mistrust of vaccines [11,23]. Digital platforms played a significant role in shaping public perceptions, with studies highlighting the impact of misinformation on social media users, online forum participants, and those engaging with user-generated content [12,14,21,22,26,28-30]. Moreover, specific populations such as Serbian adults, American women, racial minority individuals, students, public health professionals, and essential workers were reported as being particularly affected or targeted by misinformation campaigns [15,25,27,31,32].

### Impacts of Misinformation on Public Health Outcomes

#### Identified Negative Impact

The findings presented many negative effects of misinformation on public health (Table 2). One primary consequence was the impact on health care professionals, who faced challenges in discerning accurate information, leading to disruptions in routine decision-making and care practices [24]. The public was also affected, with misdirected responses and increased reliance on unproven remedies, indicating missed opportunities for effective epidemic control [10]. Misinformation significantly disrupted health and risk communication, contributing to social unrest and heightened anxiety [3]. It also directly impacted public health measures, as evidenced by lower intent to accept COVID-19 vaccines [11], reduced adherence to official health guidelines [25], and noncompliance with basic preventive measures such as handwashing [4].

The spread of misinformation resulted in decreased public trust in science [14], undermining the effectiveness of public health messaging [22] and leading to increased vaccine hesitancy [27,29,31,32]. This hesitancy was further exacerbated by the promotion of antivaccine propaganda, posing a barrier to achieving herd immunity [30]. The extent of the impact of misinformation was also evident in the public’s mental health, with reports of increased anxiety, suicidal thoughts, and distress [2], as well as in overall public attitudes toward the pandemic [26] and changes in public attitudes toward vaccines, which became increasingly negative over time [23].

**Table 2:** Impact, strategies, and effectiveness of interventions in addressing misinformation.

Study, year	Identified negative impact	Measured outcomes	Potential contributing factors	Intervention strategies	Intervention method	Platform or channel	Effectiveness metrics	Reported effectiveness
Datta et al [24], 2020	Misinformation prevalence among health care professionals	Survey responses	Social media, infodemics	Information regulation, training for accurate information identification	Training, guidelines	Official websites, media	Misinformation reduction, decision-making	Increased awareness of need for accurate information
Hou et al [10], 2020	Misdirected public responses, reliance on unproven remedies	Search trends, purchasing behaviors	Delayed communication, misinformation amplification	Public awareness enhancement, timely misinformation response	Official guidelines, rumor clarification	Government health commission, World Health Organization	Public behavior correlation with trends	Adoption of protective measures, reduction in rumor-driven behavior
Moscadelli et al [3], 2020	Disruption in health or risk communication	Fake news shares, misinformation proportion	Cognitive biases, digital literacy deficiencies	Health literacy improvement, social media policy reinforcement	Social media analysis, campaigns	Social media	Engagement in fake versus verified news	Increased strategies for discerning false news and stress reduction
Loomba et al [11], 2021	Reduced COVID-19 vaccine acceptance	Vaccine acceptance intent	Misinformation exposure, trust in sources	Misinformation impact assessment	Survey, exposure analysis	Web-based panel, social media	Vaccination intent change	Lower vaccination intent due to misinformation
Nowak et al [4], 2021	Nonadherence to sanitary recommendations, increased fears	Handwashing frequency, disinfectant use	Gender, education, living environment	Accurate information dissemination	Web-based behavior assessment survey	Social media, message boards	Adherence to preventive measures	Increased adherence to sanitary recommendations
Scholz et al [21], 2021	Link between information and quarantine measure reactions	Quarantine concern level, compliance	Demographics, media effectiveness	Continuous media reception for risk communication	Loudspeaker announcements, leaflets	Television, radio, internet	Quarantine measure approval	High acceptance or support limiting disease transmission
Teovanović et al [25], 2020	Reduced adherence to health guidelines	Prevention behavior frequency, vaccination intentions	Belief in conspiracy theories	Countering the negative impacts of irrational beliefs	Factual information and debunking	Social media, digital platforms	Engagement in evidence-based health behavior	Importance of strategies in health behavior modification
Agley et al [14], 2021	Lower trust in science due to misinformation belief	Preventive behavior intentions	Political orientation, demographics	Use of infographic to explain the scientific process	Infographic exposure	Web based (Prolific platform)	Trust in science, misinformation belief	Small increase in trust; indirect misinformation effect
Bokemper et al [22], 2022	Reduced public health messaging effectiveness	Beliefs and social distancing scales	Liberty values endorsement, conspiracy theory belief	Community protection–focused strategies	Random message interventions	Web-based survey platform	Social distancing attitudes, intentions	Improved attitudes and intentions toward distancing
Ghaddar et al [2], 2022	Reduced vaccination intent, increased mental health issues	Vaccination intent, conspiracy belief acceptance	Fake news exposure	Promotion of credible sources, debunking	Public campaigns, educational outreach	Television, radio, official channels	Trust in sources, vaccination intent	Increased trust in information sources
Kim et al [26], 2022	Misdirection in pandemic management	Vaccination intention, mandate compliance	Cultural nonconformity, misinformation spread via social media	Tailored communication to misinformed groups	Categorization, analysis	Social media	Intervention specificity and reach	Strategy specificity and misinformation reduction
Kumar et al [13], 2022	Misinformation increase related to vaccine events	Reddit discussion analysis	Media releases, community dynamics	Countering misinformation, engaging skeptics	Accuracy assessment, evidence-based discussion	Reddit, web-based spaces	Theoretical belief shift, vaccine uptake	Effectiveness proposed based on analysis
Xue et al [23], 2022	Negative public attitudes toward vaccines	Public attitude change, engagement metrics	Information source impact, emotional response	Use of fact-checking messages	Fact-checking posts, collaboration	Facebook	Public attitude change, engagement metrics	Positive role of third-party fact checkers
Mourali and Drake [28], 2022	Increased confusion, uncertainty, and negative attitudes toward health topics	Attitudes toward masking, truth objectivity, argument strength, source competence, sharing intentions	Extended debates undermining truth objectivity	Debunking misinformation	Web-based randomized study	Reddit-like social media simulation	Masking disposition, truth objectivity, sharing intentions	Correcting misinformation improved masking disposition and reduced sharing but waned with repeated exposure
Verma et al [15], 2022	Increased anxiety, especially among specific demographics	Anxiety levels from Twitter (subsequently rebranded X) data	Prior anxiety, exposure to misinformation	Misinformation exposure limitation, direct interventions	Algorithmic feed adaptation	Social media	Anxiety increase after sharing misinformation	Anxiety increase among misinformation sharers
AL-Jalabneh [29], 2023	Increased vaccine hesitancy	Frequency of misinformation themes	Social media misinformation spread	Media literacy campaigns	Educational campaigns, collaboration	Various media channels	Vaccine attitudes, misinformation reduction	Improved vaccine acceptance and trust
Gruzd et al [30], 2023	Vaccine hesitancy promotion	Proportion of misinformation in content	Social media algorithms	Misinformation removal, evidence-based content promotion	Platform moderation, messaging	Facebook, YouTube	Post or account removals, provaccine content prevalence	Partial success in misinformation reduction
Huang et al [27], 2022	Increased vaccine hesitancy	Vaccine hesitancy scale scores	Infodemic, misinformation impact	Timely health education, authoritative information use	Educational campaigns, messaging	Social media, health care settings	Vaccine hesitancy reduction, willingness to change	Positive impact on vaccination willingness
Kim et al [12], 2023	Lower health guidance compliance	Trust in experts, severity perception	Misinformation exposure, political influences	Improving regulatory efforts to curb the spread of misinformation	Survey research to identify misinformation impact	Web-based survey, media analysis	Trust levels, compliance rates	Improved discernment of false or real news, reduced stress and depression related to the pandemic
Kosiyaporn et al [31], 2023	Vaccine hesitancy due to misinformation	Vaccine acceptance rates, trust levels	Risk perception, discerning true information	Infodemic management, vulnerable group prioritization	Surveys, interviews	Web-based channels, volunteer networks	Vaccine acceptance, misinformation discernment	Increased discernment of true or false information correlated with increased vaccine acceptance
Ugarte and Young [32], 2023	Increased hesitancy and misinformation	Web-based discussion engagement	Web-based misinformation, study limitations	Community peer support	Peer leader educational engagement	Facebook groups	Misinformation on social support posts	Reduction in misinformation posts, social support increase

#### Measured Outcomes

The studies highlighted the challenges that individuals and communities faced in navigating the pandemic amid a flood of misinformation (Table 2). It was reported that misinformation significantly impacted health care professionals, leading to discomfort, distraction, and difficulty in discerning accurate information. This impact affected decision-making and routine practices [24]. The public’s response was manifested by changes in search behaviors and purchasing patterns, reflecting the influence of rumors and celebrity endorsements [10]. It was reported that “fake news” significantly affected the information landscape, skewing the perception of truth versus lies [3]. Hesitancy was reported in intent to receive COVID-19 vaccines across demographic groups [11,27,31]. The misinformation also altered health behaviors, such as handwashing and the use of disinfectants, and influenced preventive behavioral intentions [4,14]. It was also reported that misinformation affected public adherence to COVID-19 prevention, risk avoidance behaviors, and vaccination intentions [25].

The communication strategies during quarantine, public trust and engagement with authorities, and compliance with quarantine measures were influenced by the level of concern, which was shaped by misinformation [21]. It was reported that misinformation led to changes in social distancing and mask wearing [22]. Social media platforms exhibited a prevalence of antivaccine content and a focus on misinformation in web-based discussions [13,30,32]. The studies also reported that emotional and linguistic features in vaccine-related posts influenced public attitudes toward vaccines, reflecting the impact of different information sources [23]. Anxiety levels were heightened due to exposure to misinformation, especially among specific demographic groups [15]. Some of the studies (2/21, 10%) found that misinformation affected public trust in health experts and government and altered the perceived severity of COVID-19 [12,26].

#### Potential Contributing Factors

The studies identified a wide array of factors that contributed to the spread of misinformation during the pandemic (Table 2). Key among these were social media and connections with family and friends, which hastened the spread of unregulated information [24]. The issue was further compounded by delayed and nontransparent communication from health authorities, coupled with the absence of early, authoritative responses [10]. Cognitive biases, a lack of digital and health literacy, and the exploitation of social divisions also played significant roles [3]. Factors such as sociodemographic characteristics, trust in information sources, the frequency of social media use, and the nature of misinformation were important [11]. The spread of misinformation was also influenced by gender, education level, and the distinction between urban and rural living [4], as well as age, the effectiveness of media channels, the initial understanding of SARS-CoV-2, and trust in authorities, particularly in relation to quarantine measures [21]. Contributing factors included beliefs in conspiracy theories, cognitive intuition, an overestimation of COVID-19 knowledge, and susceptibility to cognitive biases [25], alongside political orientation and religious commitment [14]. Public behavior was also shaped by concerns about government infringement on personal freedoms [22]. Finally, exposure to fake news and conspiracy stories [2], cultural attitudes toward government mandates, and the spread of misinformation through social media were noted [26].

### Public Health Communication Strategies and Their Effectiveness

#### Intervention Strategies

The studies highlighted the critical role of effective public health communication strategies in addressing COVID-19 misinformation (Table 2). This included a range of approaches such as enhancing health literacy and reinforcing social media policies against fake news [3], along with using fact checking and empathetic communication to debunk misinformation [23]. The importance of timely and accurate information dissemination, particularly through social media, was also noted as a crucial component for authoritative communication [4,10,27].

In addition, several studies advocated for tailored communication approaches. These approaches involve targeting specific misinformed subgroups [26], using infographics to clarify scientific processes [14], and focusing on community protection while reframing reckless behaviors [22]. Essential strategies included training health care professionals to accurately identify credible information, alongside implementing media literacy campaigns and prioritizing groups considered vulnerable in public communication [24,29,31]. Engaging skeptics, particularly vaccine skeptics, through interventions was reported as essential [13,32], with an emphasis on debunking misinformation, promoting credible information sources, and reducing exposure to misinformation [2,15,28,30].

#### Intervention Methods

The included studies reported various intervention methods to combat misinformation. Key strategies included the use of credible sources [3,24,27], the implementation of targeted campaigns, and the integration of digital technologies such as social media tools and algorithmic analyses (Table 2) [4,10,15]. Educational efforts, ranging from basic loudspeaker announcements to sophisticated web-based educational tools and infographics, were also reported to be effective [2,14,21,29]. The importance of engaging the public through surveys, randomized interventions, and peer discussions was noted [11,22,31,32]. Fact checking, in partnership with third-party organizations and through internal processes, was highlighted as crucial, along with the need for empathetic communication [23]. Finally, some of the studies (2/21, 10%) showed the importance of identifying predictors and using analytical models to refine strategies and better understand public sentiment [26,28].

#### Platform or Channel for Communication

The studies reported that a diverse array of platforms and channels played a crucial role in effective communication during the COVID-19 pandemic (Table 2). Digital and social media platforms, such as Facebook, Reddit, and YouTube, were extensively used to disseminate facts and counter misinformation, as noted by numerous studies (8/21, 38%) [3,4,13,15,23,26,30,32]. Government websites and official channels, alongside health care settings, were also acknowledged for their value in providing reliable and accurate information [10,24,27,29]. Traditional media forms, including television, radio, and print, were found to be crucial in reaching wide audiences [2,21]. Web-based platforms designed for research and surveys, such as Prolific, played a key role in gauging public perceptions and addressing misinformation [11,14,22,28]. Furthermore, community networks and personal communications were identified as essential, particularly in village health volunteer networks and through engagement with health professionals and academics, demonstrating remarkable effectiveness in local communities and areas with limited digital access [25,31].

#### Effectiveness Metrics and Reported Effectiveness

In studies on public health communication during the pandemic, effectiveness metrics focused on reducing misinformation and improving health behaviors (Table 2) [13,24,25,27,29,31,32]. Detailed engagement metrics included tracking interactions with verified versus fake news, changes in vaccination intent, and shifts in public attitudes toward vaccines over time [3,11,23]. Unique metrics such as internet search trends correlating with public behavior, adherence to health guidelines, and the impact of misinformation on mental health were also explored [4,10,15,28]. Studies such as that by Gruzd et al [30] analyzed social media for misinformation removal and provaccine content. The reported effectiveness of interventions such as fact checking and clear communication varied across the studies, influencing vaccine attitudes and trust in science to varying degrees [11,14,23]. Some of the studies (8/21, 38%) pointed to increased public support for measures such as quarantine, emphasizing the role of community engagement [21,22], but also noted challenges in maintaining long-term effectiveness and addressing various reactions such as anxiety in response to misinformation [2,13,15,25,26,28]. These studies, often based on computational analyses, existing literature, and theoretical models, highlighted the complex, multifaceted nature of public health communication during the pandemic [3,4,30].

### Recommendations, Gaps, and Future Directions

#### Recommendations for Addressing COVID-19 Misinformation

The included studies recommended a comprehensive approach that included strategic public health communication, educational initiatives, and policy adaptation (Table 3) [2,24]. Key themes included effective information regulation and enhancing discernment skills among health care professionals as well as the general public [2,24], while strategies included considering platform-specific and demographic-focused approaches to combat misinformation [3,31]. Governmental leadership and international coordination were considered crucial [10], and educational strategies were recommended to focus on improving health literacy and researching misinformation inoculation [4,14,25]. Public health messaging and web-based moderation policies were deemed effective [13,22], and technological interventions and comprehensive policy making were recommended [15,30]. Methodological research to understand extended debates and debunking techniques was emphasized [26,28], as well as tailored communication and messaging strategies [11,12,21,27,29] (Table 3).

**Table 3:** Overview of recommendations, research gaps, and future directions in misinformation management.

Study, year	Recommendation	Specifics of recommendation	Identified gaps	Proposed future research or action
Datta et al [24], 2020	Develop training for information discernment in health care	Focus on skills for identifying and validating medical information in crises	Difficulty discerning authentic versus nonauthentic information; misinformation prevalence on social media	Formulate guidelines for medical information dissemination; enhance crisis communication skills; ethical training in information validation
Moscadelli et al [3], 2020	Strengthen strategies against misinformation in digital media	Enforce policies against fake news; develop demographic-specific communication and health literacy programs	Persistence of fake news; echo chambers on social media; low health literacy and misinformation susceptibility	Conduct research on countering fake news; enhance anti-misinformation measures on social platforms; develop targeted demographic interventions; evaluate health literacy programs
Hou et al [10], 2020	Enhance governmental risk communication and international coordination	Improve transparency and timeliness in risk communication; control misinformation; promote science-backed behaviors	Lack of timely advice for personal protection; inadequate early risk communication; missed opportunities for epidemic control	Assess the impact of government communication on public behavior; study the role of international organizations in outbreak response; develop international partnership strategies
Loomba et al [11], 2021	Adopt targeted communication strategies for vaccine misinformation	Counter misinformation with specific messaging strategies, including altruistic and scientific clarification	Lack of real-world social media research; variable impact of misinformation across demographics	Conduct social media–based studies on vaccine misinformation; establish causal relationships between misinformation types and vaccination intent; tailor public health communication for social media
Scholz et al [21], 2021	Diversify and localize communication strategies for health information	Use various media for rapid communication; address informational needs across demographics; use localized methods in rural settings	Uncertain role of health authorities; evolving media preferences during crises; variable effectiveness in information dissemination	Establish pre-event credibility of health authorities; study media habits in crises; assess long-term behavioral changes after quarantine; evaluate alternative communication methods
Nowak et al [4], 2021	Implement educational initiatives for better public understanding of preventive measures	Focus on accurate information communication and increasing public adherence to preventive measures	Challenges in public adherence to measures; susceptibility to misinformation	Conduct research on communication strategies to increase adherence; focus on demographic-specific interventions; explore psychological factors influencing public responses
Teovanović et al [25], 2020	Develop strategies to mitigate the effects of irrational beliefs and conspiracy theories	Explore and counter distrust in institutions and political cynicism; use factual corrections and debunking techniques	Reliance on self-reported data; lack of cognitive ability control; non-representativeness of sample	Investigate psychological factors affecting health behaviors; create targeted interventions; include observed behaviors in future studies for robust findings
Agley et al [14], 2021	Advance research into strategies for misinformation inoculation	Investigate the efficacy of truthful messaging about scientific processes to combat misinformation	Limited experimental research on misinformation’s behavioral effects	Conduct experimental studies testing various methods of communicating scientific processes; focus on misinformation impacts
Bokemper et al [22], 2022	Promote public health messaging to reshape social distancing perceptions and collective responsibility	Reframe social distancing in public messaging; emphasize the importance of collective protection	Uncertainty about which message elements are most effective; observed attitudinal changes not matched by behavioral changes	Dissect effective elements of public health messages; develop strategies to convert attitudes into behaviors; conduct long-term study on message impact
Kumar et al [13], 2022	Advocate for public health messaging and web-based moderation to address misinformation	Develop tailored communication strategies; engage with committed antivaccine groups; introduce verified-information tags	Challenges in changing beliefs of antivaccine individuals; moderating web-based information	Target interventions at vaccine skeptics; enhance web-based moderation policies; evaluate the effectiveness of these strategies
Kim et al [26], 2022	Focus on methodological research to identify specific misinformation types	Investigate distinct misinformation strains (eg, “vaccine chip” vs “vaccine poison”)	Misalignment between initial misinformation categories and their public health impact; lack of detailed study on antivaccine misinformation	Conduct research on different antivaccine misinformation subtypes; focus on underrepresented communities for comprehensive insights
Huang et al [27], 2022	Strategic communication and interventions for vaccine hesitancy	Target health care providers and the public with educational campaigns	Need for improved information dissemination; lack of health care provider communication training	Research effective communication strategies; create platforms to combat misinformation; design targeted interventions
Xue et al [23], 2022	Comprehensive communication strategies to combat vaccine misinformation	Design posts that will better engage the public; balance negative misinformation with empathetic communication	Underexplored impact of various information sources on vaccine attitudes; emotional responses to health communication not fully understood	Study the influence of information sources on public engagement; investigate emotional appeals in health communication; develop strategies for credible sources to enhance social media influence
Verma et al [15], 2022	Technological and educational interventions for misinformation-related anxiety	Use machine learning and social media data for anxiety detection; use health literacy initiatives	Challenges related to privacy, the First Amendment; limitations in fact-checking resources; unexplored causal relationships	Explore ethically compliant technological interventions; develop efficient resource allocation policies; create inclusive educational programs; conduct extensive studies on psychological and sociodemographic impacts
Mourali and Drake [28], 2022	Extended research on social media debates and debunking techniques	Examine the effectiveness of humor and infographics in debunking; test “prebunking” strategies	Generalizability of findings to other platforms; effectiveness of debunking in extended debates	Quantify occurrence of extended debates; investigate the impact of message elements and sources; examine consequences of engaging with misinformation spreaders
Ghaddar et al [2], 2022	Enhance critical thinking and credibility in public health communication	Promote trusted information sources; evaluate social media content critically	Effectiveness of communication strategies; understanding of belief drivers	Conduct longitudinal studies on public behavior and attitude changes; perform research on social media content engagement
Kim et al [12], 2023	Develop communication strategies to counter misinformation and enhance public trust	Focus on enhancing public trust and compliance with health guidelines	Limited research on misinformation mechanisms	Investigate interventions to mitigate misinformation effects; study impact on public trust and guideline compliance
Gruzd et al [30], 2023	Policy- and platform-based interventions for misinformation management	Strengthen misinformation policies; launch proactive public health campaigns	Inconsistent policy enforcement; persistence of echo chambers	Conduct research on the effectiveness of platform interventions; develop strategies against echo chambers
AL-Jalabneh [29], 2023	Strategic and educational interventions to reduce vaccine hesitancy	Media literacy campaigns; government-media collaboration to improve health literacy	Insufficient health literacy; widespread misinformation on social media	Adopt a collaborative approach to combat misinformation; conduct effectiveness studies of interventions; develop long-term health literacy improvement strategies
Kosiyaporn et al [31], 2023	Strategic public health communication and infodemic management	Enhance infodemic management; target groups considered vulnerable with specific communication strategies	Lack of large-scale surveys that include noninternet users; limited exploration of misinformation–vaccine acceptance relationship	Monitor misinformation trends; implement fact checking and legal actions; develop communications to debunk myths
Ugarte and Young [32], 2023	Strategy adaptation and research in public health contexts	Apply community peer support and educational engagement to combat misinformation	Small sample size; high engagement skewness; selection bias in Facebook users	Extend intervention duration; increase sample size; focus on factual information dissemination; consider a broader demographic

#### Identified Gaps in Addressing Misinformation

The studies highlighted several gaps in managing COVID-19 misinformation and public health communication. Challenges included distinguishing authentic information from misinformation, the persistence of fake news, and the presence of echo chambers in social media networks (Table 3) [3,24,30]. Timely, actionable advice for personal protection and effective risk communication during the early stages of the pandemic was lacking [10]. Research limitations included a lack of real-world simulation, leading to challenges in generalizability [11,25,26]. There was insufficient understanding of the role of health authorities as trusted sources, media preference during crises, and the effectiveness of information dissemination in different regions [2,21]. Challenges arising from legal and ethical considerations, resource limitations, disparities in education access, and insufficient exploration of the relationship between misinformation and vaccine acceptance were also noted [15,31,32] (Table 3).

#### Proposed Future Research and Actions

Future research directions included developing guidelines for medical information dissemination, enhancing crisis communication skills among health care professionals, and creating targeted interventions based on demographics (Table 3) [3,13,24,30]. Evaluating the impact of governmental and international organization communications, conducting research within social media settings, and analyzing the impact of misinformation more accurately were recommended [10,11]. Studying media habits during crises, examining long-term behavioral changes after quarantine, and dissecting the influential aspects of messages were suggested [14,21,25]. Investigating psychological factors, evaluating emotional appeals in health communication, and developing strategies for credible sources to enhance their social media influence were proposed [23,26]. Ethically and legally compliant technological interventions, efficient resource allocation policies, and extensive studies on psychological impacts were recommended [15]. Mourali and Drake [28] proposed quantifying extended debates, studying message elements and sources, and exploring “prebunking.” Longitudinal studies, research on user engagement with social media content, and interventions to mitigate misinformation effects were highlighted [2,12,32]. Finally, the studies suggested a holistic approach involving collaboration among companies, governments, and users; continuous monitoring of misinformation trends; regular fact checking; legal actions against sources of misinformation; and specific communications to debunk myths [29,31] (Table 3).

## Discussion

### Principal Findings

Our study underscores the profound influence of misinformation during the COVID-19 pandemic, particularly in shaping public responses. Misinformation, primarily propagated through social media, led to widespread misconceptions about the severity of COVID-19 infection, triggering public confusion, reluctance to adhere to health guidelines, and increased vaccine hesitancy. This phenomenon significantly impacted vaccine uptake rates. Gallotti et al [33] highlighted the simultaneous emergence of infodemics alongside pandemics, underlining the critical role of both human and automated (bots) accounts in spreading information of questionable quality on platforms such as Twitter. The authors introduced an Infodemic Risk Index to measure the exposure to unreliable news, showing that the early stages of the COVID-19 pandemic saw a significant spread of misinformation, which only subsided in favor of reliable sources as the infection rates increased [33]. This emphasizes the complex challenge of managing infodemics in tandem with biological pandemics, necessitating adaptive public health communication strategies that are responsive to evolving information landscapes. Our findings resonate with historical observations in public health crises, evidenced by studies on the Zika virus outbreak [34], polio vaccination efforts in India and Nigeria [35], and the Middle East respiratory syndrome outbreak [36]. Similar patterns of misinformation were also noted in the H1N1 pandemic and the Ebola outbreak. These instances highlight the critical need for clear, proactive communication strategies to effectively manage misinformation and guide public understanding and responses.

The review also reveals a predominant focus on digital misinformation, underscoring the necessity to comprehend the impact of traditional media and word-of-mouth communication in spreading misinformation. While studies such as that by Basch et al [37] have started to address this gap, there is a clear need for more extensive research, particularly on the long-term effects of misinformation on public health behaviors after a pandemic. This shift toward credible information, as observed by Gallotti et al [33], signals an opportunity for future research to explore capitalizing on changing information consumption patterns in public health messaging. Such observations are crucial for developing effective communication strategies, highlighting the necessity of integrating infodemic management with pandemic response efforts to mitigate misinformation effects and guide public behavior appropriately. The disparity in the effectiveness of misinformation mitigation strategies points to the need for a nuanced understanding of how misinformation evolves over time. Studies, such as that by Vijaykumar et al [38], highlight the challenges in countering rapidly changing misinformation narratives on digital platforms. Further investigation into the effectiveness of fact checking across different cultures and demographics, as suggested by Chou et al [39], is essential for developing better strategies to combat misinformation in diverse settings.

This review found that various factors, including delayed communication from health authorities, cognitive biases, sociodemographic characteristics, trust in official sources, and political orientation, played a significant role in the spread of misinformation during the pandemic. These findings align with similar observations in other studies. Eysenbach [40] emphasized the importance of trust in government agencies and health care providers in shaping individuals’ beliefs and their willingness to share accurate information during public health crises. In addition, Pennycook and Rand [41] highlighted how political beliefs and affiliations can influence people’s interpretation of information, thus impacting their acceptance or rejection of official guidance during public health crises. The study by Gallotti et al [33] also highlighted the differentiated roles of verified and unverified users on social media in propagating COVID-19–related information. Their analysis shows that verified users began to point more toward reliable sources over time, hinting at the potential of leveraging social media influencers and verified accounts in directing public attention to factual and scientifically verified information [33].

These insights indicate the critical need for dynamic public health strategies that are adaptable and actionable, aimed at curtailing misinformation through education and technology. It is essential to incorporate digital literacy and clear, audience-specific messaging to effectively counter misinformation, a strategy that has proven successful in health crises beyond the COVID-19 pandemic; for example, during the H1N1 pandemic, targeting specific audience segments with tailored messages significantly improved public understanding and guideline compliance [42]. Likewise, during the Ebola outbreak, proactive and transparent strategies were key in dispelling rumors and building trust in public health authorities [43]. These approaches, based on an understanding of the target audience’s concerns and media habits, are consistent with our findings where digital literacy and targeted messaging played a critical role in mitigating COVID-19 misinformation effects. Such strategies are vital not only for immediate crisis response but also for fostering long-term resilience in public health communication, helping to enable the public to distinguish credible information from misinformation, with the ultimate goal of enhancing public health outcomes and trust in health authorities.

In examining the authoritarian responses to the pandemic, particularly in Brazil and Turkey, it is evident that leadership tactics significantly contributed to societal polarization and misinformation. Leaders in these countries used the crisis to suppress dissent and consolidate power, often spreading misinformation and underreporting COVID-19 cases, thereby exacerbating public mistrust and confusion [5]. Similarly, a study of communication strategies across countries with high rates of infection emphasized the variation in political leaders’ approaches, where strategies ranged from science-based communications to ideologically influenced messaging [6]. The study highlighted the potential for political leaders to influence public health responses through their communication tactics, further impacting public behavior and trust in health guidelines [6]. In certain situations, the integration of political ideology with public health messaging, as observed in countries such as the United States, Brazil, India, and the United Kingdom, not only perpetuated misinformation but also intensified societal rifts [5,6]. This highlights the paramount role of leadership in navigating public health crises; for instance, in the United States and Brazil, political leaders’ approaches to the COVID-19 pandemic—characterized by mixed messaging on mask wearing and social distancing—contributed to public confusion and a politicized response to the pandemic. Similarly, the initial underestimation of the virus’s impact in India and the United Kingdom’s delayed lockdown response serve as examples of how political decisions can shape public health outcomes and trust in health authorities, emphasizing the profound impact of aligning political views with public health communication [5,6]. In addition, the initial reluctance of the World Health Organization to endorse mask wearing, social distancing, and handwashing, followed by a later reversal of these recommendations, exemplifies the challenges and confusion created by global health leadership during the early stages of the pandemic [44]. Such shifts in guidance contributed to the global spread of misinformation, further complicating public health responses and trust in international health authorities [6]. These approaches, based on an understanding of the target audience’s concerns and media habits, are consistent with our findings that digital literacy and targeted messaging played a critical role in mitigating COVID-19 misinformation effects. Such strategies are vital not only for immediate crisis response but also for fostering long-term resilience in public health communication, helping to enable the public to distinguish credible information from misinformation, with the ultimate goal of enhancing public health outcomes and trust in health authorities. Applying the MEGA framework in practical settings could revolutionize public health communication, offering a model for how technology can be harnessed to tackle misinformation more effectively. By processing massive graph data sets and accurately computing infodemic risk scores, MEGA supports the development of targeted communication strategies and interventions. Its approach to preserving crucial feature information through graph neural networks signifies a leap forward in optimizing learning performance, underscoring the framework’s utility in crafting evidence-based policies and initiatives to effectively combat misinformation. This emphasizes the importance of integrating advanced technological solutions, such as MEGA, into public health strategies to enhance the precision and effectiveness of infodemic management [19]. The integration of social media literacy into public health strategies is emphasized as essential by Ziapour et al [7], suggesting that a populace equipped with advanced media literacy skills exhibits greater resilience against misinformation.

Our study reveals the profound impact of the COVID-19 infodemic, which extended beyond public health and eroded trust in health institutions and government authorities. This decline in trust contributed to societal polarization, mirroring the effects seen in the Ebola outbreak, where misinformation led to notable repercussions [45,46]. Further research, similar to that conducted on the Zika outbreak by Basch et al [37], is needed to understand the long-term effects of misinformation on societal cohesion and trust. Addressing this evolving landscape of misinformation requires dynamic and adaptable public health policies. These strategies should integrate insights from various methodologies, using both digital and traditional media for greater reach and impact, drawing lessons from the successful strategies deployed during the H1N1 pandemic, such as those highlighted by Chou et al [39].

Our study advocates for a collaborative approach, uniting governments, the private sector, and the public in a concerted effort to combat misinformation, highlighting the importance of joint action in this global challenge. This approach should include continuous monitoring of misinformation trends, implementing regular fact checking, taking legal action against sources of misinformation, and developing specific communications to debunk myths. Similar findings have been reported in studies addressing misinformation related to the Zika virus [34,47], yellow fever [48], and Ebola [49], emphasizing the importance of a holistic strategy involving all stakeholders [50].

### Limitations

The review has several limitations to consider. First, there is a temporal limitation because it included only studies published between December 2019 and September 2023, potentially excluding more recent research that could have offered additional insights. Second, the reliance on specific databases (MEDLINE [PubMed], Embase, and Scopus) as the primary sources for data might have led to the omission of pertinent studies that are not indexed in these databases. Third, the study’s sole focus on research articles may have excluded valuable insights from other scholarly works such as conference papers, theses, case studies, and gray literature. Finally, it is important to acknowledge that the study’s restriction to English-language publications may have excluded valuable research conducted in other languages. While efforts were made to review the available literature comprehensively, omitting non-English sources could limit the breadth and depth of the findings. Recognizing these limitations, future endeavors should aim to expand the scope of research beyond these constraints, incorporating a more diverse range of sources, languages, and real-world interventions to enrich our understanding of, and response to, misinformation.

### Conclusions

The results of this review emphasize the significant and complex challenges posed by misinformation during the COVID-19 pandemic. It shows how misinformation can have a wide impact on public health, societal behaviors, and individual mental well-being. The findings highlight the critical role of effective public health communication strategies in addressing the infodemic. It is essential that these strategies are not only targeted and precise but also adaptable and inclusive, ensuring that they are relevant to diverse demographic and sociocultural contexts.

The review also emphasizes the need for ongoing collaborative research efforts to further explore the nuances of the misinformation spread and its consequences. This requires cooperation among health authorities, policy makers, communication specialists, and technology experts to develop evidence-based approaches and policies to combat misinformation.

Furthermore, the review highlights the importance of refining public health communication strategies to keep up with the ever-changing nature of misinformation, especially in the digital realm. It advocates using advanced technology and data-driven insights to enhance the reach and impact of health communication. By combining scientific rigor, technological innovation, and empathetic communication, these strategies can contribute to building public trust, promoting health literacy, and creating resilient communities capable of recognizing and countering misinformation.

In summary, the lessons learned from the COVID-19 pandemic emphasize the necessity of strengthening public health communication infrastructures. This strengthening is vital for addressing the current misinformation crisis and preparing for future public health emergencies. Implementing these recommendations will play a crucial role in shaping a more informed, aware, and health-literate global community better equipped to confront the challenges posed by misinformation in our increasingly interconnected world. Furthermore, future research directions should explore integrating advanced large language models with frameworks similar to MEGA. This exploration will bolster automated fact checking and infodemic risk management, contributing to more effective strategies in combating misinformation in public health communication.

## Appendix

Multimedia Appendix 1 PRISMA-ScR (Preferred Reporting Items for Systematic Reviews and Meta-Analyses extension for Scoping Reviews) guidelines.

## Abbreviations

MEGA: machine learning–enhanced graph analytics
PRISMA-ScR: Preferred Reporting Items for Systematic Reviews and Meta-Analyses extension for Scoping Reviews
